# Awareness, perceptions and barriers to digital dentistry adoption among dentists in India: A questionnaire survey

**DOI:** 10.6026/973206300220577

**Published:** 2026-01-31

**Authors:** Jinal Patil, Monali Shah, Ruchi Raval, Anand Bhalodi, Jay Chetan Shah, Sangita Bhalodi

**Affiliations:** 1 Department of Periodontology, K.M. Shah Dental College and Hospital, Sumandeep Vidyapeeth Deemed to be University, Vadodara, Gujarat, India; 2 Private Dental Practitioner, Restore Dental Treatment Centre, Rajkot, Gujarat, India; 3 Dentist, Smile Design Dentistry, Florida, USA; 4 Restore Dental Treatment Centre, Rajkot, Gujarat, India

**Keywords:** 3D Printing, CAD/CAM, digital dentistry, cone beam computed tomography

## Abstract

Studies in India report general awareness of digital dentistry among dental practitioners; however, its clinical use remains inconsistent. There are significant gaps in practical understanding, particularly of advanced digital tools. Assessing dentists' attitudes, barriers and identifying gaps in awareness are therefore essential to integrate digital dentistry in clinical practice. A cross-sectional, questionnaire-based online survey was conducted among 408 dentists by the Department of Periodontology, K. M. Shah Dental College and Hospital, Sumandeep Vidyapeeth Deemed to be University, Vadodara, Gujarat, India. The survey assessed knowledge and use of digital radiography, CBCT, smile design software, intraoral scanners and 3D printing. Digital radiography showed the highest adoption, followed by CBCT, while advanced digital tools had lower awareness and usage. Overall awareness was moderate, with adoption constrained by cost, integration issues and data security concerns, emphasizing the need for enhanced training in CBCT and oral radiology.

## Background:

Digital dentistry involves using digital technologies in dental practice [1]. Digital technologies significantly change diagnosis, treatment planning, restorative procedures and education [2]. Key digital technologies include intraoral scanners and cameras, CAD/CAM systems for design and milling, digital radiography, CBCT, MRI and CT scans, 3D printing, AI-enhanced planning, augmented and virtual reality and digital practice management. Traditional dentistry has been essential for oral healthcare, but has several limitations compared to modern digital methods. Conventional dentistry often relies on invasive treatments that may cause overtreatment rather than preventive care [3]. They are also time-consuming, involve multiple manual steps and are prone to errors [4]. Modern methods have shown greater healing outcomes after surgical endodontic and management of dental caries [5]. Compared to conventional methods, digital workflows reduce chair-side time and manual errors [6]. Digital approaches also improve patient comfort and overall clinical outcomes [7]. Digital radiography and CBCT have enhanced diagnostic capabilities in routine dental care [8]. Intraoral scanners and CAD/CAM systems enable precise digital impressions and restorations [9]. Three- dimensional printing supports surgical guides, prosthetics and customized dental appliances [10]. The transition to digital methods in dental medicine, fuelled by electronic health records, is recognized as one of the significant developments of the 21st century. This shift addresses both current and future challenges in dental and oral healthcare. Despite benefits, high costs and limited training restrict widespread adoption [11]. Studies in India report variable awareness and inconsistent use among dental practitioners [12]. It indicates that while there is general awareness, gaps remain in practical understanding, particularly with advanced tools. This highlights the need for more hands-on training and exposure. Understanding dentists' perceptions is also essential to improve the adoption of digital dentistry. Therefore, it is of interest to identify and address gaps in awareness of digital dentistry among dentists in India.

## Materials and Methods:

A questionnaire-based study was conducted by the Department of Periodontology, K.M. Shah Dental College and Hospital, Sumandeep Vidyapeeth Deemed to be University, Vadodara, Gujarat, India. The purpose of the web-based survey was to evaluate awareness and practices related to digital dentistry. The questionnaire was self-developed and cross-validated by eight experts from K.M. Shah Dental College and Hospital. The survey used a closed-ended questionnaire format (Annexure 1) and was designed with a template from Google Forms (Google Inc., USA). The participants consisted of professionals with BDS and MDS degrees. The study was conducted over a period of three months, following approval from the Institutional Ethics Committee (IEC). A digital link to the participant information sheet and consent form was generated and sent out via email, message, WhatsApp, phone or in person. Once all participants completed the questionnaire, the data were entered into an Excel sheet for statistical analysis. A single examiner reviewed all forms. This questionnaire had two parts. The first part collected demographic details from the respondents. The second part asked questions about clinical practices involving digital radiography (X-rays, CBCT), smile design software, 3D printing and intraoral scanners. Baseline characteristics were described using the mean and standard deviation for quantitative variables and percentages for categorical variables. The sample size was determined after a pilot study, using a power of 80%, α = 0.05 and a standard deviation. Simple random sampling was completed and a total of 408 individuals participated in the study.

## Results:

A total of 408 dentists participated in the study. All data were entered and organized in Microsoft Excel (Version 2013) and subsequently analysed using the Statistical Package for the Social Sciences (SPSS, Version 16.0; IBM, USA). Descriptive statistics were applied and results were expressed as frequencies and percentages for categorical variables. Continuous variables were summarized using mean and standard deviation (SD). Demographic distribution by age consisted of the largest proportion of the 20-29 years age group (47.5%), followed by 30-39 years (43.4%) and 40-49 years (9.1%). Overall, the gender distribution was relatively balanced, with 52.2% male and 47.8% female participants. A gender difference was observed across age groups, with females predominating in the 20-29 years group (60.3%). Males were more represented in the 30-39 years (62.7%) and 40-49 years (67.6%) groups (Table 1). The majority of respondents (98.0%) reported an increase in cases involving digital dentistry over the last three years (Table 2). Awareness and familiarity with intraoral scanners were reported by 91.6% of participants. Knowledge of the advantages of digital impressions over conventional methods was high, with 93.9% of participants being fully informed. Adoption of digital technologies varied by tool. Digital radiography (X-rays, CBCT) is used extensively by 89.7% of respondents, with another 9.1% using it to some extent. CAD/CAM is used regularly by 86.8%, occasionally by 10.0% and not at all by 3.2%. Digital smile design software is used often by 87.5%, occasionally by 9.3% and never by 3.2%. 3D printing shows similar uptake, with 87.7% using it extensively, 8.1% partially and 4.2% not yet integrated. A significant majority (89.0%) perceived the major barrier of digital dentistry as expensive. Despite this, 95.3% agreed with charging higher fees for digital workflows. Confidence in performing digital workflows for implant planning was expressed by 93.4% of respondents. Continuing education engagement was notable, with 87.5% attending multiple courses in the past year and only 3.9% reporting no exposure. Most participants (95.1%) strongly believed that digital dentistry improves patient outcomes, while 93.6% expressed satisfaction with the integration of digital technologies into their practice. CBCT was primarily utilized in Implantology (76.5%), followed by periodontology (15.0%) and Endodontics (8.6%). For intraoral scanners, the most common indication was guided implant surgery (53.4%), followed by digital smile design (42.9%) and aligners (3.7%).

## Discussion:

Digital dentistry includes a wide range of technologies that serve clinical, diagnostic and educational purposes. Digital radiography, cone-beam computed tomography (CBCT) and advanced optical imaging improve diagnostic accuracy. Computer-aided design and computer-aided manufacturing (CAD/CAM), along with 3D printing, enable precise fabrication of dental restorations and appliances [13]. Artificial intelligence (AI) and machine learning are increasingly used for disease detection and treatment planning. They also help in automated image segmentation and aesthetic outcome prediction, thereby improving diagnostic reliability [14]. Fully integrated digital workflows streamline clinical procedures, reduce chair-side time, increase efficiency and enhance patient comfort and satisfaction [15]. Beyond diagnosis and treatment, digital educational tools are widely valued by both students and faculty. However, a preference for conventional assessment methods remains, indicating that a hybrid teaching approach may produce better learning outcomes [16]. Despite these benefits, key challenges persist and need to be overcome. Key barriers include the need for strong clinical validation, difficulty integrating different data sources and ongoing training requirements as systems rapidly evolve [17]. The present study employed a questionnaire-based survey methodology, yielding findings that provided several noteworthy insights. Respondents demonstrated a marked preference for intraoral scanners, digital radiography, CAD/CAM systems, smile design software and 3D printing technologies. Overall, these modalities were perceived as valuable tools that enhance patient outcomes and facilitate greater treatment precision. Many practitioners reported confidence in implementing digital workflows and high satisfaction with clinical outcomes. Multiple studies have been conducted across various countries, involving diverse participant groups, yielding varied results. Hall *et al.* [18] conducted an online assessment through social media from November 2022 to March 2023. They focused on Egyptian dentists from different specialities, experience levels and health sectors. The findings indicated that about half of the participants had moderate knowledge and awareness of digital dentistry. Meanwhile, 75.9% perceived its value highly. Awareness was significantly lower among female dentists and those in government clinics. It was higher among faculty staff, urban practitioners and certain specialities. In 2021, Nayakar *et al.* [19] surveyed to evaluate knowledge, awareness and practices regarding digital dentistry, specifically in intraoral scanning, 3D printing and CAD/CAM. This study examined its implications in dental practice among postgraduate students and practitioners in India. The results showed high awareness (96.67%) of CAD/CAM, with a lack of knowledge identified as the main barrier. Teaching faculty had a better understanding than private practitioners and students. In another questionnaire-based survey at Justus Liebig University Giessen, the views of undergraduate dental students on two digital Prosthodontic training modules were evaluated. The modules focused on the digital analysis of tooth preparations and the fabrication of CAD/CAM restorations. Students reported positive experiences regarding usability, educational value and learning motivation. Most students preferred instructor-led assessment and feedback over purely digital evaluation. More than 90% expressed willingness to use intraoral scanners in future clinical practice. These findings suggest that digital tools serve as effective complements to traditional teaching methods rather than complete replacements [20]. Two recent online surveys were conducted in 2025. In Saudi Arabia, a cross-sectional survey of dentists found higher digital adoption among Generation X practitioners, strong support for integrating digital dentistry into undergraduate education and identified limited awareness, education, leadership and clinical evidence as major barriers [21]. In the United States, an online survey of board-certified Periodontists reported routine use of CBCT and implant planning tools, with adoption varying by experience and practice type, while cost, complexity and uncertain clinical benefits remained key barriers [22]. A questionnaire-based study by Shah *et al.* in in 2025 at Ahmedabad was done with the aim to assess the perception, awareness, and attitude of dental practitioners toward digital dentistry. It was conducted among 120 general dental practitioners using a 12-item closed-ended online survey. Moderate knowledge was reported by 83 participants, while 116 showed a positive attitude toward digital dentistry. 63 practitioners preferred digital techniques for better accuracy, whereas 59 preferred conventional methods due to cost- effectiveness. Awareness was maximum for intraoral scanners (76 participants), while the satisfaction with CAD-CAM prostheses was remarkable [23]. Few of these findings are comparable to those observed in the current study. Together, and these studies reveal considerable gaps in awareness and adoption of digital dentistry. They emphasize the need for strong, system-level strategies to address these gaps. Improving awareness and use of digital dentistry relies on three main strategies. First, education must be reinforced through professional development, hands-on training and the integration of technology into dental curricula. Second, barriers like high equipment costs and a lack of technical support should be tackled through subsidies and shared resources. Finally, promoting a culture of innovation can increase motivation and demand for digital change in dental practice [24, 25-26].

## Conclusion:

Digital technologies are reshaping dentistry by improving accuracy, efficiency and patient outcomes. Despite clear benefits, adoption remains uneven due to gaps in training, readiness and infrastructure. Advancing education and standardized digital workflows will be key to fully realizing the potential of digital dentistry.

## Figures and Tables

**Table 1:** Age group-wise Gender distribution of participants

**Age Group (In years)**	**Male**	**%**	**Female**	**%**	**Total**
20 to 29	77	39.7	117	60.3	194
30 to 39	111	62.7	66	37.3	177
40 to 49	25	67.6	12	32.4	37
Total	213	52.2	195	47.8	408

**Table 2:** Comparative analysis of participants' responses

**Questionnaire / Items**		**Over all**	
		N = 408	%
Do you think the cases of digital dentistry have increased in the last 3 years?	Yes	400	98.03
	No	2	0.49
	Can't Say	6	1.47
How familiar are you with digital intraoral scanners for capturing impressions?	Familiar	374	91.6
	Neutral	31	7.59
	Not familiar at all	3	0.73
Have you incorporated digital radiography (X-rays, CBCT) in your practice?	Yes, extensively	366	89.7
	Yes, to some extent	37	9.06
	No, not yet	5	1.22
Do you use CAD/CAM technology for designing and manufacturing dental restorations (crowns, bridges)?	Yes, regularly	354	86.76
	Occasionally	41	10.04
	No, not at all	13	3.18
How often do you utilize digital smile design software for treatment planning in cosmetic dentistry?	Often	357	87.5
	Sometimes	38	9.31
	Never used	13	3.18
Have you adopted 3D printing for fabricating dental models or surgical guides?	Yes, extensively	358	87.7
	Yes, to some extent	33	8.08
	No	17	4.16
If no, do you plan to invest in adopting 3D printing in the next 1-2 years?	Yes	9	2.2
	NO	1	0.24
	Not Sure	7	1.71
Do you think digital dental practice is expensive?	Yes	363	88.97
	NO	35	8.57
	Not Sure	10	2.25
Are you aware of the advantages of digital impressions over traditional impressions using impression materials?	Fully aware	383	93.87
	Somewhat aware	21	5.14
	Not aware at all	4	0.98
Do you agree to charge more from the patients for the digital dental workflow?	Strongly Agree	389	95.34
	Can't Say	13	3.18
	Don't Agree	6	1.47
How confident are you in using digital workflows for implant planning and placement?	Confident	381	93.38
	Neutral	20	4.9
	Not confident at all	7	1.71
Have you attended continuing education courses or training related to digital dentistry in the past year?	Yes, multiple courses	375	87.5
	Yes, one course	17	4.16
	No, none	16	3.92
Do you believe digital dentistry improves patient outcomes compared to traditional methods?	Strongly believe	388	95.09
	Neutral	17	4.16
	Strongly disbelieve	3	0.73
How satisfied are you with the integration of digital technologies in your dental practice?	Satisfied	382	93.62
	Neutral	24	5.88
	Dissatisfied	2	0.49
In your dental practice, where do you use CBCT most commonly?	Endodontics	35	8.57
	Implantology	312	76.47
	Periodontology	61	14.95
From below mentioned which is the most useful indication of an Intraoral scanner?	Aligners	15	3.67
	Digital smile design	175	42.89
	Guided implant surgery	218	53.43
